# Thermo-Compression of Thermoplastic Chitosan Films Reinforced with Microcrystalline Cellulose for Antibacterial Food Packaging Application

**DOI:** 10.3390/polym17182460

**Published:** 2025-09-11

**Authors:** Prasong Srihanam, Theeraphol Phromsopha, Aphidech Sangdee, Nuanchai Khotsaeng, Pham Ngoc Lan, Yodthong Baimark

**Affiliations:** 1 Biodegradable Polymers Research Unit, Department of Chemistry and Centre of Excellence for Innovation in Chemistry, Faculty of Science, Mahasarakham University, Maha Sarakham 44150, Thailand; prasong.s@msu.ac.th (P.S.); theeraphol.p@msu.ac.th (T.P.); 2 Department of Biology, Faculty of Science, Mahasarakham University, Maha Sarakham 44150, Thailand; aphidech.s@msu.ac.th; 3 Microbiology and Applied Microbiology Research Unit, Faculty of Science, Mahasarakham University, Maha Sarakham 44150, Thailand; 4 Faculty of Science and Health Technology, Kalasin University, Kalasin 46230, Thailand; nuanchai.ko@ksu.ac.th; 5 Faculty of Chemistry, University of Science, Vietnam National University-Hanoi, 19 Le Thanh Tong Street, Phan Chu Trinh Ward, Hoan Kiem District, Hanoi 10000, Vietnam; phamngoclan49@gmail.com

**Keywords:** thermoplastic chitosan, microcrystalline cellulose, biocomposites, thermo-compression, antibacterial food packaging

## Abstract

Thermoplastic chitosan/microcrystalline cellulose (TPC/MCC) composite films were prepared by thermo-compression and are reported here for the first time. L-lactic acid (LLA) was used as a plasticizer in the formation of TPC. TPC films with varying LLA contents and the TPC/MCC composite films with different MCC contents were produced for evaluation. The physicochemical, mechanical, and antibacterial properties of the thermo-compressed TPC and TPC/MCC films were characterized. LLA enhanced thermal stability and crystallinity, improved film flexibility, and reduced the water solubility of the chitosan matrix. Incorporation of MCC further improved mechanical properties and decreased water dissolution. Tensile testing showed that the addition of 5 wt% MCC increased maximum tensile strength by 82% and Young’s modulus by 124%. All TPC and TPC/MCC films exhibited antibacterial activities against both Gram-positive *Staphylococcus aureus* and Gram-negative *Escherichia coli*. Antibacterial efficacy decreased as MCC content increased to 20 wt%. These thermo-compressed TPC/MCC films can be tailored to display a range of properties by adjusting the contents of LLA and MCC, making them well suited for antibacterial food-packaging applications.

## 1. Introduction

Plastic waste has become a major pollution issue worldwide, particularly that from single-use food packaging. Plastic waste from polypropylene, polyethylene, and polystyrene products, which take hundreds of years to biodegrade, is classified as non-biodegradable polymers [1]. Therefore, using biodegradable polymers to replace their non-biodegradable counterparts is an alternative that can help reduce the pollution problem of plastic waste. Biodegradable polymers can degrade by simple hydrolysis before being further broken down into CO_2_ and water by microorganisms [2,3].

Biodegradable polymers can be divided into two main types: petroleum-based and bio-based polymers. Poly(ε-caprolactone) (PCL), poly(butylene adipate-*co*-terephthalate) (PBAT), and poly(butylene succinate) (PBS) are examples of biodegradable petroleum-based polymers. In contrast, biodegradable bio-based polymers include poly(lactic acid) (PLA), polyhydroxyalkanoates (PHAs), and natural materials such as proteins (including silk fibroin and keratin) or polysaccharides (including cellulose, starch, chitosan, and alginate). Biodegradable and bio-based polymers show a lower carbon footprint compared to their petroleum-based counterparts [1,4,5,6]. Therefore, food packaging produced from biodegradable and bio-based polymers has been a topic of widespread interest and has been extensively investigated [4,6,7].

Chitosan is synthesized through partial deacetylation of chitin, the principal constituent of crustacean exoskeletons, such as those of crabs and shrimp [8,9]. As one of the most abundant biopolymers found in nature, chitosan exhibits remarkable film-forming capabilities, biodegradability, biocompatibility, non-toxicity, and antimicrobial properties [10,11,12,13,14]. Most chitosan-based products are produced from chitosan solutions using evaporation processes. This method limits the ability to scale up the production of chitosan-based products for industrial applications. Chitosan typically undergoes thermal decomposition before it reaches its melting point, thus limiting the forming of chitosan-based products using conventional melt processing techniques [15]. Thermoplastic chitosan (TPC) is a modified variant of chitosan that has been plasticized, enabling it to be molded via melt processing methods. Few reports have been published on the fabrication of TPC films that utilize a thermo-compression technique [15,16,17]. L-Lactic acid (LLA) has been used as an effective non-volatile plasticizer in the preparation of TPC [17].

However, chitosan film is often limited by inadequate mechanical strength and a tendency to swell [8]. Combinations of chitosan and cellulose, as well as their composites, have been shown to improve mechanical properties, antibacterial effectiveness, biocompatibility, formability, and metal ion adsorption [8,9,18,19,20]. The chitosan/cellulose composites have been fabricated in various forms, such as fibers, films, and particles, which have been utilized in agriculture, the food sector, biomedical applications, and the extraction of hazardous metals, pigments, and dyes from wastewater or contaminated water [8,9]. Microcrystalline cellulose (MCC) is an abundant, sustainable, biodegradable, and cost-effective filler for chitosan [8,9]. In addition, MCC has high mechanical properties [21] and substantially enhances the formulation of various polysaccharide films, increasing their strength. The formation of hydrogen bonds between polysaccharides and MCC facilitates the stress transfer from external forces [22,23].

To our knowledge, there is currently no published literature on the melt processing of TPC/MCC composites suitable for scaling up to industrial applications. This paper focuses on the preparation of TPC by plasticizing chitosan with LLA and the fabrication of TPC/MCC composite films through a thermo-compression process. The effects of LLA and MCC contents on the physicochemical, mechanical, and antibacterial properties of the composite films were analyzed.

## 2. Materials and Methods

### 2.1. Materials

Chitosan powder with a 94% degree of deacetylation and a viscosity of 300 cps (measured at 20 °C from 2 wt% chitosan solution in 1 wt% acetic acid aqueous solution) was purchased from Sinudom Agriculture Ltd., Part. (Surathani, Thailand). The chitosan powder was sieved through a 200-mesh sieve. L-lactic acid (LLA) solution with a concentration of 88 wt% was purchased from Purac (Rayong, Thailand). Microcrystalline cellulose (MCC) with an average particle size of 50 µm was purchased from Acros Organics (Geel, Belgium). Glycerol (QReC brand, 99.5%) purchased from Smart Science Co., Ltd. (Pathum Thani, Thailand).

### 2.2. Preparation of Thermoplastic Chitosan/MCC Composite Films

**Figure 1:**
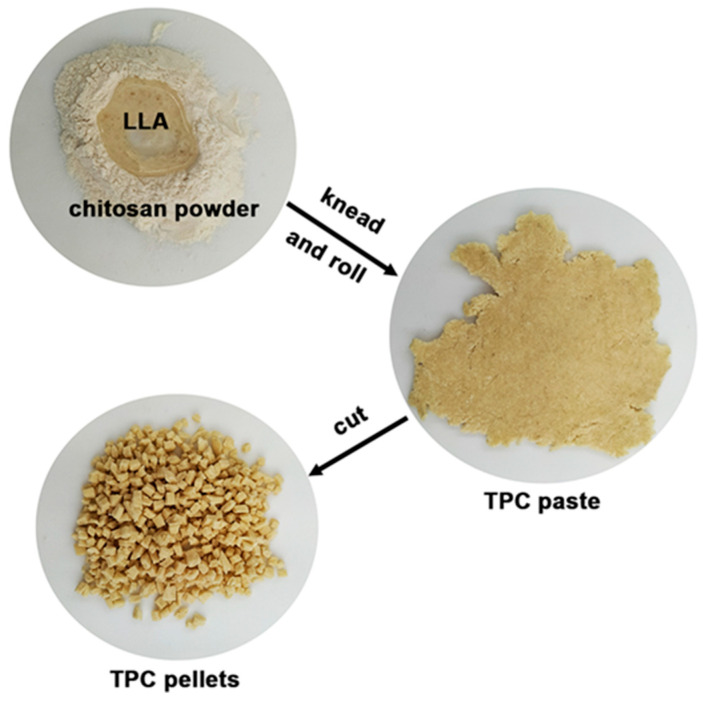
Preparation process of TPC pellets.

To prepare thermoplastic chitosan (TPC) films, chitosan powder was kneaded and rolled together with an LLA solution until a uniform mixture was achieved. This TPC paste was then cut into pellets using scissors, as illustrated in Figure 1. The LLA contents of 50 wt%, 60 wt%, and 70 wt% relative to the weight of chitosan were examined. Preliminary experiments showed that when the LLA content was less than 50 wt%, the resulting TPC paste was not homogeneous. When the LLA content was more than 70 wt%, the TPC paste became homogeneous; however, this resulted in some LLA oozing. The TPC pellets were subjected to thermo-compression at a temperature of 120 °C for 5 min, during which a pressure of 5 MPa was applied using an Auto CH Carver hot-press machine (Wabash, IN, USA). After this process, the films were cooled with cool plates while maintaining the same compressed force of 5 MPa for an additional 5 min.

To prepare TPC/MCC composite films, a mixture of chitosan and MCC powder, along with an LLA solution, was kneaded and rolled until a uniform consistency was achieved. This TPC/MCC paste was then cut into pellets. The LLA content was kept constant at 50 wt%, based on the weight of chitosan. The MCC contents of 5 wt%, 10 wt%, and 20 wt% based on the weight of chitosan were investigated. The TPC/MCC composite pellets were then thermo-compressed under the same conditions previously described. The TPC and TPC/MCC films were stored at 25–30 °C and a relative humidity of 50–60% for a duration of 14 days before characterization [24,25].

### 2.3. Characterization of TPC and TPC/MCC Composite Films

The chemical structures of each sample were examined using an Invenio-S Fourier transform infrared (FTIR) spectrometer (Bruker, Karlsruhe, Germany) equipped with attenuated total reflection (ATR) diamond. The ATR-FTIR spectra were recorded in a wavenumber range of 500 to 4000 cm^−1^ with an accumulation of 32 scans at a spectral resolution of 4 cm^−1^.

The thermal decomposition behaviors of the samples were evaluated using an SDT Q600 thermogravimetric analyzer (TGA, TA Instruments, New Castle, DE, USA). A 100 mL/min nitrogen flow rate was employed. The TGA analysis involved heating the sample (5–10 mg) from 50 to 800 °C at a rate of 20 °C/min.

The phase morphology of cryo-fractured surfaces of film samples was investigated using a JSM-6460LV scanning electron microscope (SEM, JEOL, Tokyo, Japan) running at 15 kV acceleration voltage. The film samples were fractured using liquid nitrogen. Before performing the SEM scanning, a thin layer of gold was deposited on the film samples using a sputter coating technique.

The crystalline structures of the film samples were analyzed using a D8 Advance wide-angle X-ray diffractometer (XRD, Bruker, Karlsruhe, Germany) operating with CuKα radiation at 40 kV and 40 mA. The scan velocity was 3°/min.

The tensile properties of the film samples (70 mm × 10 mm) were evaluated using an LY-1066B universal testing machine (Dongguan Liyi Environmental Technology Co., Ltd., Dongguan, China) at 25 °C, employing a 100 kg load cell. The initial gauge length for the experiments was set at 50 mm. A crosshead speed of 50 mm/min was applied. The tensile properties were calculated by averaging five measurements.

The moisture content of the film samples was determined as follows. The film samples (20 mm × 20 mm) were first weighed (W_1_) prior to being dried at 105 °C for 24 h. Subsequent to the drying process, the film samples were reweighed (W_2_). The moisture content was subsequently determined using the following equation:

Moisture content (%) = [(W_1_ − W_2_)/W_1_] × 100 (1)

The water dissolution of film samples was determined using the following method. The film samples (20 mm × 20 mm) were dried at 105 °C for 24 h before weighing (W_3_). Following the weighing process, we immersed the film samples in 50 mL of distilled water and agitated them at 100 rpm at 25 °C for 24 h. Following the dissolution process, the film samples were dried at 105 °C for 24 h and then reweighed (W_4_). The water dissolution was then calculated using the following equation:

Water dissolution (%) = [(W_3_ − W_4_)/W_3_] × 100 (2)

The water contact angle of film surfaces was measured using the sessile drop method with an OCA11 contact angle analyzer (DataPhysics Instruments, Filderstadt, Germany). Following the application of 2.5 µL of deionized water, which was deposited onto the surface from both the left and right sides of the droplet, the contact angles on the film surface were recorded and averaged after 15 s.

The film opacity of the samples was evaluated by measuring the absorbance at a wavelength of 600 nm (A_600_) using a Cary 60 UV-Vis spectrophotometer (Agilent Technologies, Victoria, Australia). The film thickness was measured by a digital micrometer (Mitutoyo, Tokyo, Japan) with an accuracy of 0.001 mm. The film’s opacity was calculated using the following equation [26,27]:

Film opacity (mm^−1^) = A_600_/X (3)

where X is the film thickness (mm).

Gram-positive *Staphylococcus aureus* DMST 2933 (*S. aureus*) and Gram-negative *Escherichia coli* ATCC 25922 (*E. coli*) were used in the antibacterial assay. Each bacterial strain was initially cultured on Mueller Hinton Agar (MHA) at 37 °C for 16–18 h. A single colony was then inoculated into Mueller Hinton Broth (MHB) and incubated at 37 °C for 4 h with shaking at 250 rpm. The bacterial suspension was subsequently adjusted to the 0.5 McFarland standard. The standardized inoculum was evenly swabbed across the entire surface of MHA plates in four directions. Sterile film samples (5 mm in diameter) were prepared by immersing them in 70% ethanol, blotting dry with autoclaved filter paper, and subsequently exposing to ultraviolet light for 10 min. These film samples were placed on the inoculated agar surface and incubated at 37 °C for 16–18 h. After incubation, the diameter of the inhibition zone around each film was measured in millimeters and recorded. The average diameter of the inhibition zone was calculated using three separate tests.

### 2.4. Statistical Analysis

The experimental data were analyzed using one-way ANOVA, followed by Duncan’s post hoc test. The results are expressed as mean ± standard deviation (SD), demonstrating statistically significant differences at *p* < 0.05.

## 3. Results and Discussion

### 3.1. The Effect of LLA Content on Properties of Thermo-Compressed TPC Films

#### 3.1.1. ATR-FTIR Analysis

**Figure 2:**
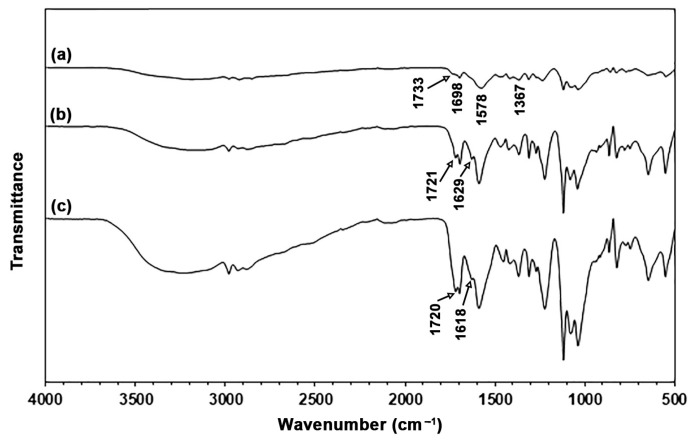
ATR-FTIR spectra of TPC films with LLA contents of (**a**) 50 wt%, (**b**) 60 wt%, and (**c**) 70 wt%.

The chemical functional groups present in the TPC films, along with the potential intermolecular interactions between the chitosan and LLA, were analyzed using ATR-FTIR spectra, as illustrated in Figure 2. The ATR-FTIR spectrum of the 50% LLA-TPC film, shown in Figure 2a, displays a broad absorption band in the range of 3000–3500 cm^−1^, which indicates the presence of O–H and N–H stretching vibrations [28]. The absorption bands in the range of 2800 cm^−1^ to 3000 cm^−1^ are attributed to C–H stretching vibrations [21]. The spectrum also exhibits a band at 1733 cm^−1^, which corresponds to the absorption of C=O stretching vibrations in lactic acid [17]. Absorption bands at 1698 cm^−1^, 1578 cm^−1^, and 1367 cm^−1^ are attributed to C=O stretching (amide I), C–N stretching and N–H bending (amide II), and C–N bending (amide III) of chitosan, respectively [29,30,31].

The ATR-FTIR spectra of the 60% and 70% LLA-TPC films, shown in Figure 2b,c, exhibit a pattern similar to that of the 50% LLA-TPC film depicted in Figure 2a. The C=O stretching vibration in lactic acid shifted from 1733 cm^−1^ to lower wavenumbers at 1721 cm^−1^ and 1720 cm^−1^ for 60% and 70% LLA-TPC films, respectively. This finding indicates that hydrogen bonding occurred between the C=O groups of LLA and the –NH_2_ groups of chitosan molecules [32]. The new absorption bands observed at 1629 cm^−1^ for the 60% LLA-TPC film and 1618 cm^−1^ for the 70% LLA-TPC film are attributed to amide I of the –CONH– groups. This effect is a result of the reaction between the –NH_3_^+^ groups of protonated chitosan and the –COOH groups of LLA [17].

#### 3.1.2. Thermal Decomposition

**Figure 3:**
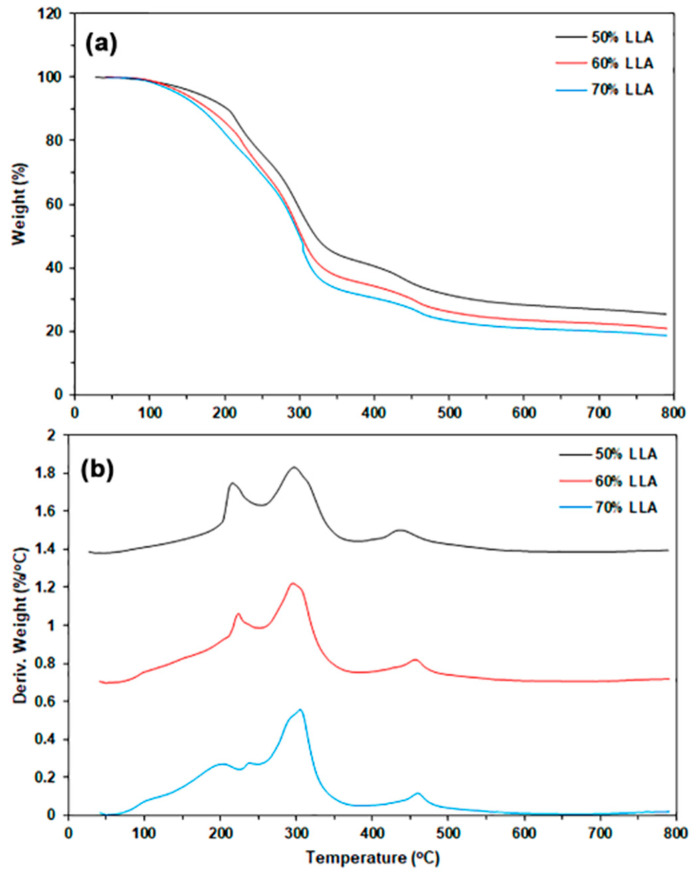
(**a**) TG and (**b**) DTG thermograms of TPC films with varying LLA contents.

The thermal decomposition behaviors of TPC films in a nitrogen atmosphere were analyzed using thermogravimetric (TG) and derivative TG (DTG) thermograms, as illustrated in Figure 3. Table 1 provides a summary of the TGA results. The TG thermograms depicted in Figure 3a reveal three distinct stages of weight loss. The first weight-loss stage occurs within a temperature range of 50–150 °C, which is attributed to the evaporation of water molecules. The second weight-loss stage, occurring between 150 °C and 250 °C, is due to the evaporation of LLA. The third weight-loss stage, spanning 250–700 °C, is associated with the thermal decomposition of chitosan molecules [33,34]. The weight losses observed in the first and second stages increased with higher LLA content, suggesting that the moisture content in the sample films also rises as the LLA content increases. The char residue at 800 °C decreased with higher LLA content because more LLA evaporated during the TGA analysis.

The DTG thermograms displayed temperature peaks at the maximum weight-loss rate (*T_max_*). As shown in Figure 3b, the three *T_max_* peaks identified in the temperature ranges of 150–250 °C, 250–400 °C, and 400–700 °C correspond to the evaporation of LLA, the cleavage of glycosidic bonds in chitosan chains, and the thermal decomposition of residual carbon in the biopolymers, respectively. The 50% LLA-TPC film shows *T_max_* peaks of 216 °C for LLA (*LLA-T_max_*) and 297 °C for chitosan (*CS-T_max_*), as reported in Table 1. Both the *LLA-T_max_* and *CS-T_max_* peaks generally shift to higher temperatures with increasing LLA content. This finding indicates that interactions between LLA and chitosan molecules have occurred, as determined by the FTIR analysis, resulting in an increase in the thermal stability of both the TPC matrix and the LLA. Further analysis is needed to explore the exact mechanisms behind these interactions and their implications for the material’s overall properties. The plasticization of chitosan with lactic acid has additionally improved the thermal stability of the chitosan matrix [17]. The lower *LLA-T_max_* peak at 204 °C was also observed for the 70% LLA-TPC film, suggesting that some aggregation of LLA molecules occurred when a higher content of LLA was utilized.

#### 3.1.3. Phase Morphology

**Figure 4:**
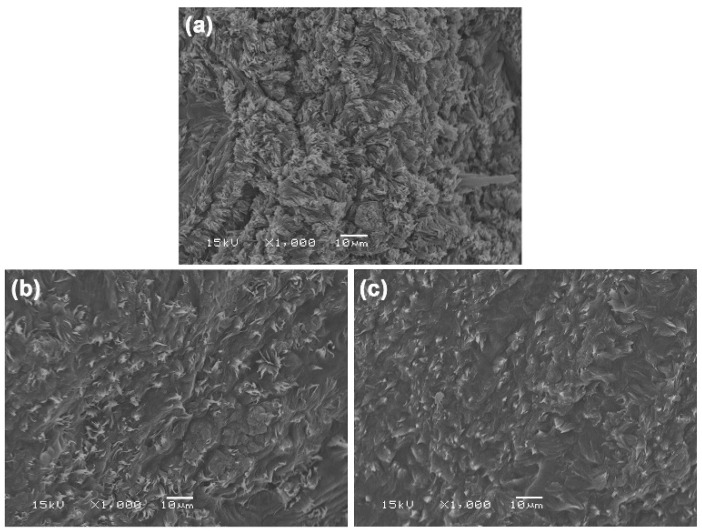
SEM images of cryo-fractured surfaces of TPC films with LLA contents of (**a**) 50 wt%, (**b**) 60 wt%, and (**c**) 70 wt%.

Figure 4 shows SEM images of cryofracture surfaces of film samples, used to study their phase morphology. At an LLA content of 50 wt%, the cryofracture surface exhibited the highest roughness. However, as the LLA content increased, the surface gradually became smoother. This trend can be attributed to LLA acting as a de-structuring agent and a non-volatile plasticizer, which disrupts hydrogen bonds between chitosan molecules and forms new, weaker hydrogen bonds between LLA and chitosan. This interaction results in a more uniform continuous phase and leads to flatter cryofracture surfaces. These findings are consistent with previous studies that indicate a higher amount of plasticizer produces more homogeneous and smoother cryo-fractured surfaces in the chitosan film matrix [35].

#### 3.1.4. Crystalline Structures

**Figure 5:**
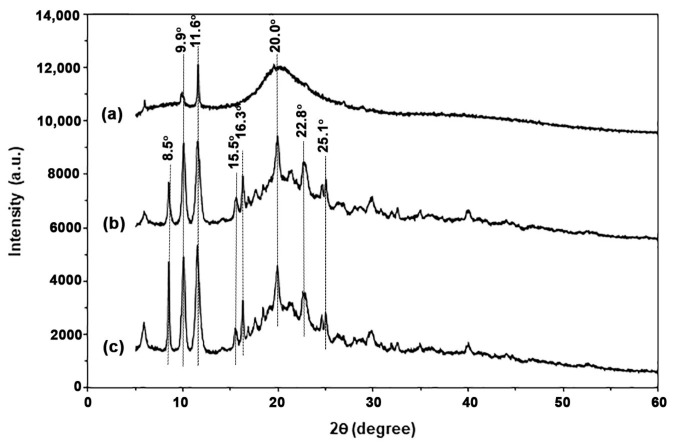
XRD patterns of TPC films with LLA contents of (**a**) 50 wt%, (**b**) 60 wt%, and (**c**) 70 wt%.

Figure 5 shows the XRD patterns of TPC films with varying LLA contents. The XRD pattern for the 50% LLA-TPC film, shown in Figure 5a, displays two peaks at 2θ = 9.9° and 20.0° assigned to chitosan crystalline [36,37]. The intensity and sharpness of the peaks at 2θ = 8.5° and 11.6° increased significantly as the LLA content increased. These peaks were proposed as an LLA-plasticized chitosan crystalline structure. It has been reported that the XRD peaks at 2θ = 9.4° and 11.4° in the chitosan film are estimated to represent a hydrated chitosan crystalline structure, resulting from the incorporation of water molecules into the chitosan crystal lattice [38]. This phenomenon may be attributed to LLA acting as a plasticizer to enhance the chain mobility of chitosan for crystallization [39]. Additionally, several XRD peaks corresponding to chitosan crystal were also observed at 60 wt% and 70 wt% LLA contents, specifically at 2θ = 15.5°, 16.3°, 22.8°, and 25.1° [40,41,42,43]. Therefore, the various XRD peaks observed in this study, apart from two XRD peaks at 2θ = 9.9° and 20.0°, indicate an LLA-plasticized chitosan crystalline structure that alters the regular arrangement of chitosan chains.

#### 3.1.5. Tensile Properties

**Figure 6:**
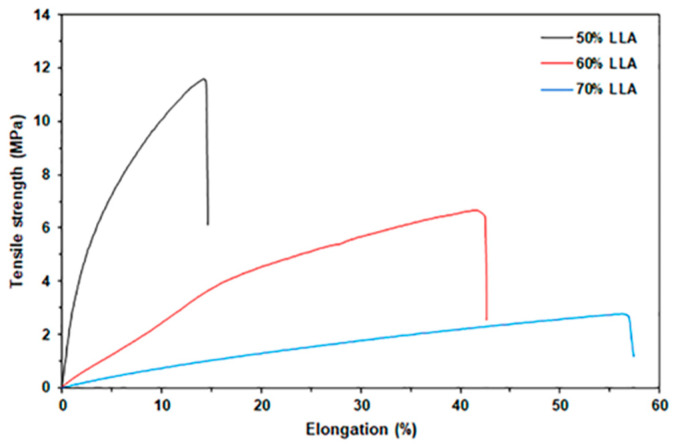
Selected tensile curves of TPC films with varying LLA contents.

Figure 6 presents tensile curves for TPC films, while Table 2 provides a summary of the tensile results. The 50% LLA-TPC film demonstrated a maximum tensile strength of 11.5 MPa, an elongation at break of 14.7%, and a Young’s modulus of 110.8 MPa. An increase in LLA content led to a significant decrease in both maximum tensile strength and Young’s modulus, while elongation at break showed a considerable increase. This finding indicates that LLA acts as an effective plasticizer for chitosan. LLA enhances the chain mobility of the chitosan by reducing the intermolecular forces between chitosan molecules, which results in greater flexibility and reduced rigidity [15,17].

#### 3.1.6. Moisture Content, Water Dissolution, and Surface Wettability

Table 3 summarizes the hydrophilicity and water dissolution characteristics of TPC films. The moisture content of TPC films increased from 2.51% to between 3.05% and 3.27% as the LLA content rose from 50 wt% to 60–70 wt%. This increase is attributed to the highly hydrophilic nature of LLA. Concurrently, the water contact angle of TPC films decreased from 60.12° to a range of 44.31° to 47.25° as the LLA content increased, indicating an enhancement in the surface wettability of TPC films. The increased hydrophilicity correlates with the increased LLA content, which can be explained by the interactions between the hydrophilic LLA plasticizer and chitosan that enhance the surface wettability of the TPC films [37]. However, the dissolution of TPC films in water decreased slightly as the LLA content increased. This reduction may be due to an increase in the crystallinity of TPC with higher LLA content, as previously observed in the XRD analysis. The higher crystallinity observed in TPC films with increased LLA content may indicate a decrease in their water solubility [36].

#### 3.1.7. Film Opacity and Antibacterial Activity

**Figure 7:**
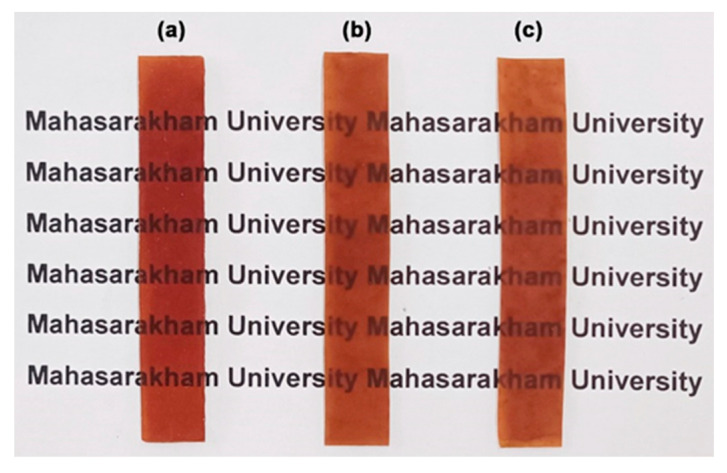
Photographs of TPC films with LLA contents of (**a**) 50 wt%, (**b**) 60 wt%, and (**c**) 70 wt%.

The effects of LLA content on the film thickness, film opacity, and antibacterial activity of TPC films are summarized in Table 4. The film thickness slightly decreased with the increase in LLA content. The plasticization effect of LLA may lead to a decrease in the melt viscosity of the TPC matrix during thermo-compression, which could result in a reduction of film thickness. The incorporation of LLA increased the opacity of TPC films. This observation may be explained by the elevated crystallinity of the film associated with the increased LLA content, as previously detailed in the XRD analysis. In general, film opacity increases as film crystallinity increases because crystalline regions scatter light more effectively than amorphous regions [44]. Figure 7 presents photographs of the TPC films, which all exhibit a clear brown color, allowing for a visible view of the characters underneath.

**Figure 8:**
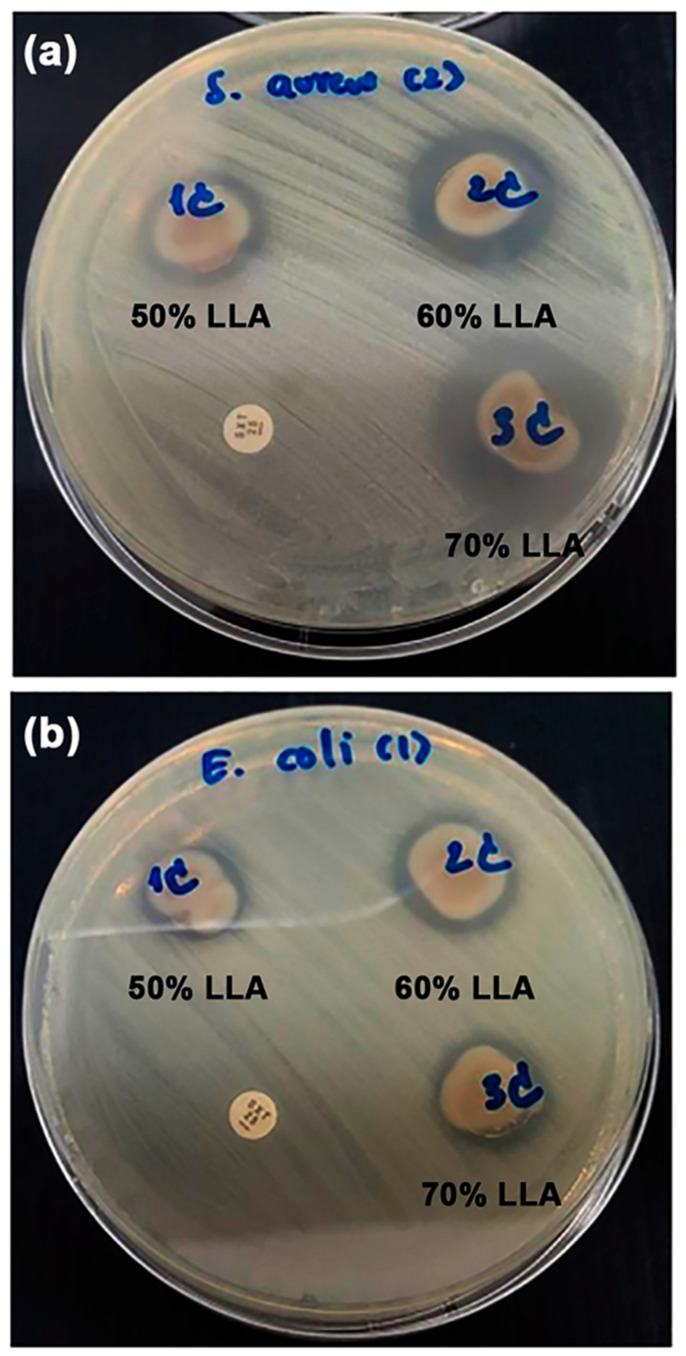
Agar disc diffusion of antibacterial activity against (**a**) Gram-positive *S. aureus* and (**b**) Gram-negative *E. coli* of TPC films with varying LLA contents.

The antibacterial activities of the film samples were assessed by measuring the growth inhibition of *S. aureus* (a Gram-positive bacterium) and *E. coli* (a Gram-negative bacterium), as illustrated in Figure 8. The antibacterial test showed a clear circle around the film samples. This circle was an inhibition zone, a place where bacterial colonies could not grow. The antibacterial activity of the film samples is directly related to the size of the inhibition zone. Inhibition zones were observed for all the TPC films. The evidence indicates that all TPC films are capable of inhibiting and preventing the growth of both bacterial types. The addition of LLA provided antibacterial properties to the chitosan films by enhancing the activity of the positively charged chitosan molecules [17].

Table 4 also summarizes the diameters of the inhibition zones in film samples. The TPC films showed an increased diameter of the inhibition zone for *S. aureus* bacteria with higher LLA content. The findings suggested that the antibacterial efficacy of the TPC films against *S. aureus* improved with higher LLA content. The inhibition zone diameter for *E. coli* bacteria in the TPC films measures between 1.8 and 2.0 mm, indicating that the LLA content did not influence the antibacterial effectiveness against *E. coli*. The process is thought to involve electrostatic interactions between the positively charged chitosan and the negatively charged bacterial cell membrane. This interaction disrupts the cell membrane, causing leakage of cellular contents and ultimately leading to cell death [45,46,47,48]. Positively charged chitosan demonstrates antibacterial activity against both *S. aureus* and *E. coli*, but it is typically more effective against *S. aureus*. This difference is commonly attributed to the structural variations in the cell walls of Gram-positive bacteria, such as *S. aureus*, and Gram-negative bacteria, such as *E. coli* [49]. *S. aureus* has a thicker peptidoglycan layer in its cell wall, which may make it more vulnerable to interactions with positively charged chitosan [48]. LLA, which is generated by lactic acid bacteria, demonstrates antibacterial properties against both *S. aureus* and *E. coli* [50,51]. However, the inhibition zone against *E. coli* in Table 4 did not increase with increasing LLA content. Thus, the antibacterial activity of TPC films is dependent on positively charged chitosan.

### 3.2. The Effect of MCC Content on Properties of Thermo-Compressed TPC/MCC Films

#### 3.2.1. ATR-FTIR Analysis

**Figure 9:**
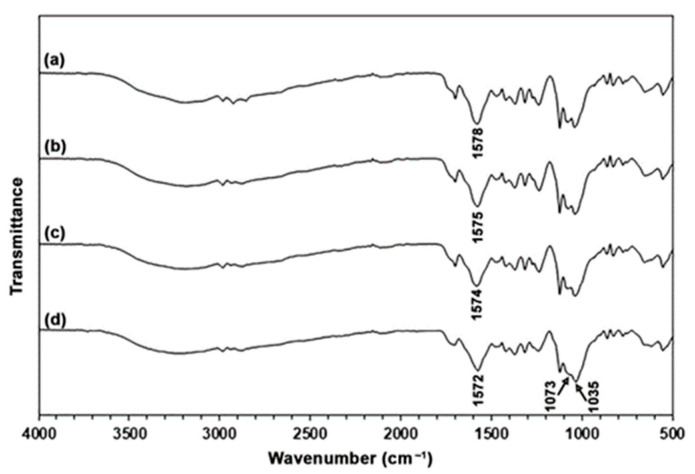
ATR-FTIR spectra of TPC films (**a**) without and with MCC contents of (**b**) 5 wt%, (**c**) 10 wt%, and (**d**) 20 wt% based on the weight of chitosan. All films contain 50 wt% LLA, based on the weight of chitosan.

In Figure 9, the ATR-FTIR spectra of TPC films containing MCC exhibit a pattern similar to that of the TPC film without MCC. This observation arises from the fact that the structures of both chitosan and MCC have similar characteristics [28]. The absorbance of bands at 1073 cm^−1^ and 1035 cm^−1^ assigned to the deformation of the glucopyranose ring of MCC [52,53] and C–O stretching vibrations of MCC [54], respectively, increased significantly as the MCC content increased compared to the absorbance of the amide II bands (1574–1578 cm^−1^) of chitosan. These results support that TPC/MCC films with varying MCC content were prepared. Additionally, the amide II band of TPC without MCC, which was observed at 1578 cm^−1^, shifted to 1575 cm^−1^ for the 5% MCC content, 1574 cm^−1^ for the 10% MCC content, and 1572 cm^−1^ for the 20% MCC content in the TPC/MCC films. The observed shifting was attributed to the interaction between the –NH_2_/–NH_3_^+^ groups of chitosan and the –OH groups of MCC [29,32].

#### 3.2.2. Thermal Decomposition

**Figure 10:**
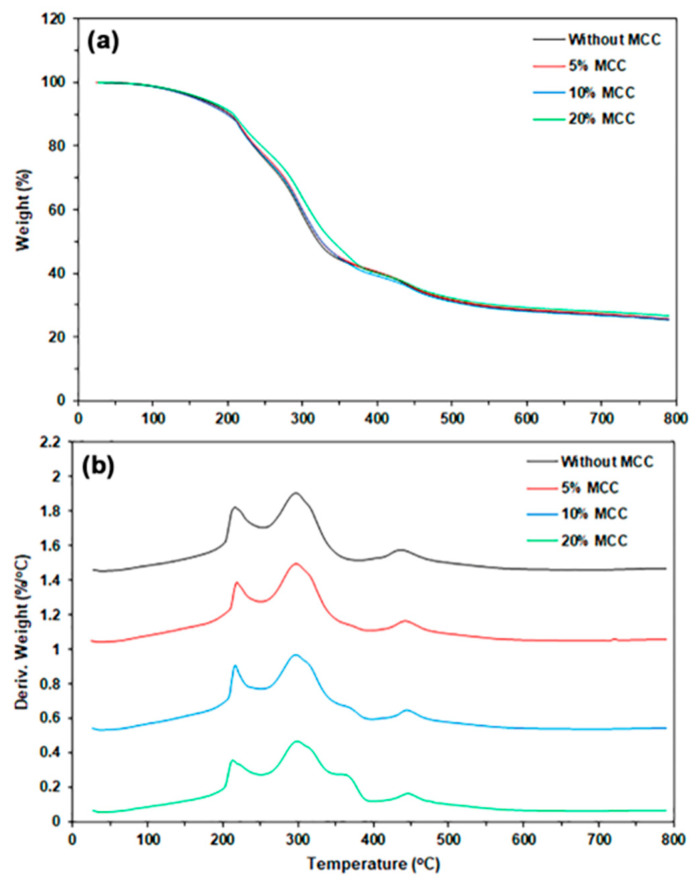
(**a**) TG and (**b**) DTG thermograms of TPC/MCC films with varying MCC contents. All films contain 50 wt% LLA, based on the weight of chitosan.

Figure 10 displays the TG and DTG thermograms for TPC/MCC films, and the TGA results are summarized in Table 5. The TG thermogram of MCC powder, illustrated in Figure S1a, shows two distinct weight-loss steps. The first step corresponds to water evaporation, occurring between 50 °C and 150 °C. The second step is associated with the depolymerization of cellulose chains, which takes place within the temperature range of 250–450 °C [55]. All TPC/MCC composite films displayed TG thermograms similar to those of the 50% LLA-TPC film. However, the weight-loss step in the temperature range of 250–400 °C shifted to a higher temperature as the LLA content increased to 70 wt%. The char residues at 800 °C for the TPC/MCC films ranged from 25.6% to 26.4%. This range was similar to the char residue value of the 50% LLA-TPC film (25.4%).

The *LLA-T_max_* peak of the TPC/MCC film shifted to a lower temperature, while the *CS-T_max_* peak shifted to a higher temperature when 20% MCC was incorporated. This finding may be explained by the formation of hydrogen bonds between chitosan and MCC, which may reduce the interactions between chitosan and LLA molecules [56]. The *MCC-T_max_* peak was clearly observed when the MCC content reached 10 wt%. The TPC/MCC films, which included 10% and 20% MCC, exhibited *MCC-T_max_* peaks at 364 °C and 366 °C, respectively. The MCC powder showed an *MCC-T_max_* peak at 355 °C in its DTG thermogram (Figure S1a). The observed shift of the *MCC-T_max_* peaks to higher temperatures suggests that chitosan interacts with MCC through hydrogen bonding, as supported by the literature [8,9].

#### 3.2.3. Phase Morphology

**Figure 11:**
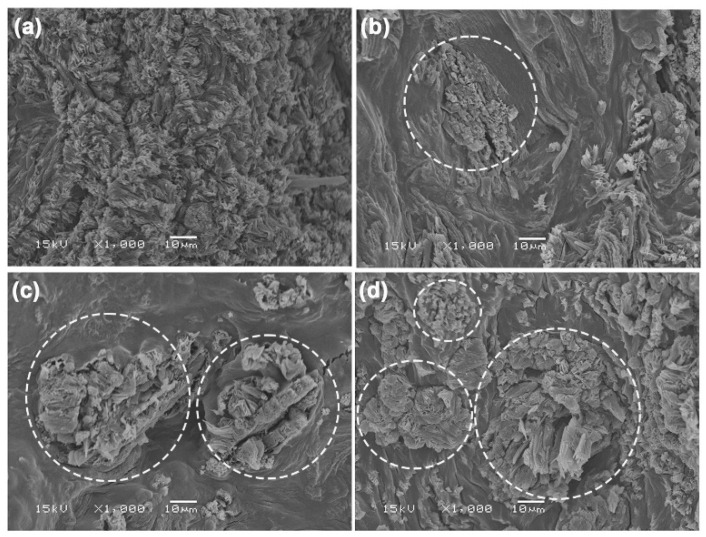
SEM images of cryo-fractured surfaces of TPC films (**a**) without MCC and with MCC contents of (**b**) 5 wt%, (**c**) 10 wt%, and (**d**) 20 wt% based on the weight of chitosan. All films contain 50 wt% LLA, based on the weight of chitosan. Some MCC particles were indicated by white circles.

The phase morphology of TPC/MCC composite films was analyzed using SEM images of cryo-fractured film surfaces in comparison to the 50% LLA-TPC film, as illustrated in Figure 11. An SEM image of the MCC particles is shown in Figure S1b, where they exhibit irregular shapes. Some MCC particles in the TPC/MCC films were distinctly visible, as highlighted by the white circles. The good phase compatibility between the MCC particles and the TPC film matrix is evident, as both materials are hydrophilic, which is indicated by their close surface contact [57,58]. This good phase compatibility suggests that the incorporation of MCC into the TPC matrix could enhance the mechanical properties of the films, potentially leading to improved performance in various applications. The agglomeration of MCC particles was observed at MCC contents of 10 wt% and 20 wt%, as shown in Figure 11c,d, respectively. This study aligns with earlier findings that suggest the addition of significant amounts of MCC leads to the aggregation of MCC particles within the chitosan matrix [59].

#### 3.2.4. Crystalline Structures

**Figure 12:**
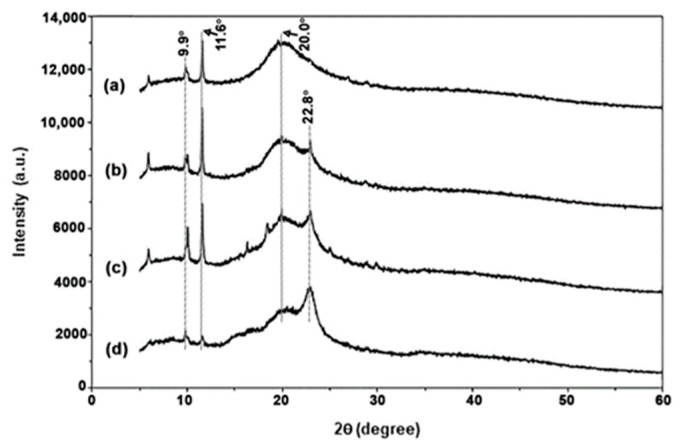
XRD patterns of TPC films (**a**) without MCC and with MCC contents of (**b**) 5 wt%, (**c**) 10 wt%, and (**d**) 20 wt% based on the weight of chitosan. All films contain 50 wt% LLA, based on the weight of chitosan.

The crystalline structures of TPC/MCC composite films were analyzed using XRD patterns, which were compared to those of the 50% LLA-TPC film, as illustrated in Figure 12. The XRD peaks for MCC powder appear at 2θ = 15.3° and 22.8°, as shown in Figure S1c, indicating a crystalline structure characteristic of cellulose type I [60,61]. Figure 12 demonstrates that the XRD peak associated with MCC at 2θ = 22.8° exhibited a significant increase in peak intensity with higher MCC content. These results suggest that TPC/MCC composite films can be prepared with varying MCC contents. Notably, the XRD peak intensities for the 50% LLA-TPC film matrix at 2θ = 9.9°, 11.6°, and 20.0° decreased significantly as the MCC content increased to 20 wt%. Furthermore, the findings indicate that the incorporation of MCC does not change the crystalline structure of the TPC films. The evidence suggests that the addition of MCC primarily influences the peak intensity rather than altering the fundamental arrangement of the crystalline phases.

#### 3.2.5. Tensile Properties

**Figure 13:**
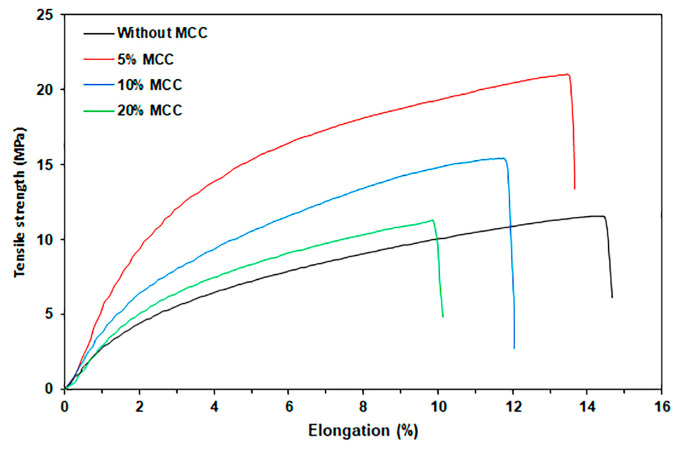
Selected tensile curves of TPC/MCC films with varying MCC contents. All films contain 50 wt% LLA, based on the weight of chitosan.

Figure 13 illustrates the tensile curves for TPC/MCC films, while Table 6 provides a summary of the tensile results. The maximum tensile strength and Young’s modulus significantly increased, while the elongation at break decreased with the addition of 5% MCC. These results suggest that the addition of MCC significantly strengthens the TPC film matrix. Interactions between chitosan and MCC enhanced effective stress transfer between the chitosan matrix and the MCC obtained from a film casting method [30]. However, when the MCC content exceeded 5 wt%, the maximum tensile strength and Young’s modulus of the TPC/MCC composite decreased. This decrease can be attributed to the aggregation of MCC particles at 10 wt% and 20 wt%, as indicated by previous SEM analysis. This aggregation reduces the available surface areas for effective stress transfer during external loading, which consequently diminishes these tensile properties [59]. MCC can serve as an effective reinforcing agent for TPC films while also contributing to reduced production costs. However, it is imperative that the mechanical properties of these composite films be tested against the standards of various food packaging materials before commercial use.

#### 3.2.6. Moisture Content, Surface Wettability, and Water Dissolution

Table 7 provides a summary of the moisture content, surface wettability, and water dissolution of TPC/MCC films. The moisture content of 50% LLA-TPC films increased significantly as the MCC content increased up to 20 wt%. The water contact angles consistently decreased (surface wettability increased) as the MCC content increased. These findings suggest that the hydrophilicity of TPC/MCC films increases with increasing MCC content. This observation can be attributed to the increased number of hydroxyl groups found in MCC, which enhances the hydrophilicity of the TPC film matrix [30]. The addition of MCC decreased the water solubility of the TPC composite films. This reduction may be attributed to an increase in the lower water-soluble MCC fraction, as well as the interactions between the MCC and the chitosan matrix. These interactions likely enhance the structural stability of the composite films, which in turn limits the overall solubility of the TPC matrix in water.

#### 3.2.7. Film Opacity and Antibacterial Activity

**Figure 14:**
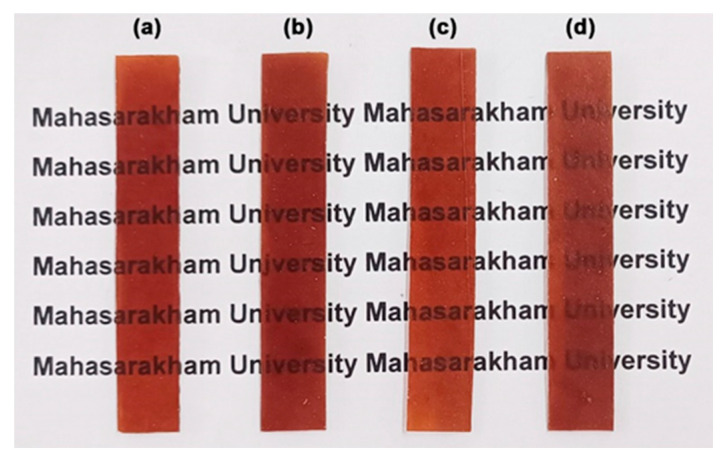
Photographs of TPC films (**a**) without MCC and with MCC contents of (**b**) 5 wt%, (**c**) 10 wt%, and (**d**) 20 wt% based on the weight of chitosan. All films contain 50 wt% LLA, based on the weight of chitosan.

Table 8 summarizes the effects of MCC content on the film thickness, film opacity, and antibacterial activity of TPC/MCC films. All films had thicknesses ranging from 0.68 mm to 0.71 mm. The addition of MCC did not influence the film thickness of the 50% LLA-TPC films. The film opacity of the TPC/MCC films increased significantly to 2.57 mm^−1^ when the MCC content was raised to 20 wt%. This increase may be due to the significant aggregation of MCC at a higher concentration [62,63]. Figure 14 shows the appearance of the TPC/MCC films, comparing the version without MCC [Figure 14a] to those with different MCC contents [Figure 14b–d]. As the MCC content reached 20 wt%, the film opacity increased; however, the letters underneath remained visible. These films can be used in packaging to maintain the visibility of product features.

**Figure 15:**
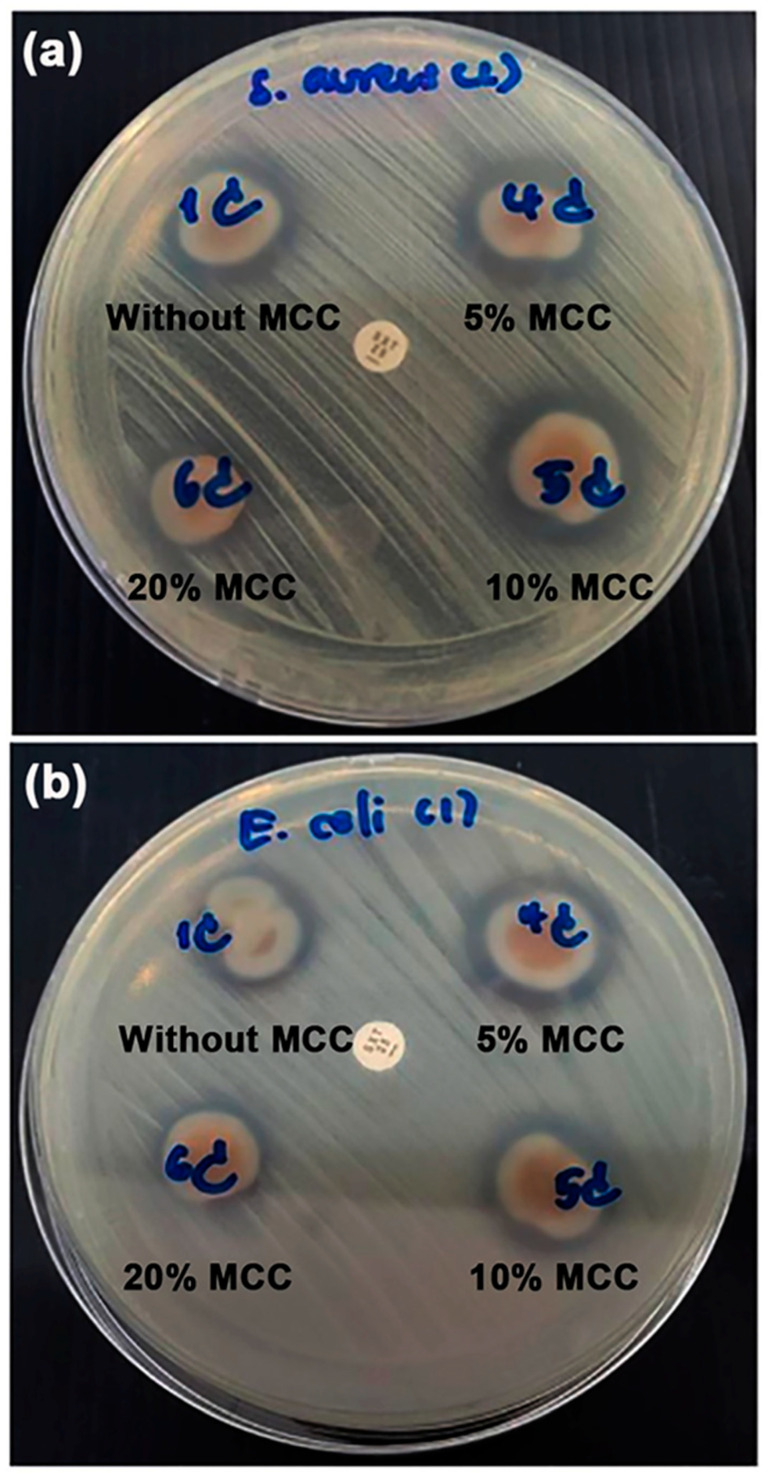
Agar disc diffusion of antibacterial activity against (**a**) Gram-positive *Staphylococcus aureus* and (**b**) Gram-negative *Escherichia coli* of TPC/MCC films with varying MCC contents. All films contain 50 wt% LLA, based on the weight of chitosan.

The antibacterial activities of the TPC/MCC composite films were evaluated by measuring the growth inhibition of *S. aureus* and *E. coli*, as illustrated in Figure 15. All TPC/MCC composite films exhibited inhibition zones for both *S. aureus* and *E. coli*, indicating their antibacterial properties. Table 8 summarizes the diameters of the inhibition zones observed in the TPC/MCC composite films. The TPC/MCC composite films containing 5 wt% and 10 wt% MCC exhibited inhibition zone diameters for both bacterial types that were nearly equivalent to those of the 50% LLA-TPC film without MCC. It has been noted that MCC does not have inherent antibacterial properties on its own. MCC can be modified or combined with other substances to develop antibacterial materials [64,65,66]. The diameters of the inhibition zones for both bacterial types decreased when the MCC content reached 20 wt%. This reduction in the antibacterial effectiveness of TPC/MCC composite films is likely attributed to the diminishing presence of the chitosan fraction as the MCC content increases.

## 4. Conclusions

TPC films plasticized with LLA and reinforced with MCC were successfully prepared using a thermo-compression technique. Strong interaction between the chitosan and LLA was found from FTIR analysis, and a new functional group was also formed from their interaction. The incorporation of LLA into the chitosan enhanced the thermal stability, crystallizability, and flexibility of the chitosan film matrix. All TPC films show antibacterial activity for both *S. aureus* and *E. coli*. The inhibition zone increased for *S. aureus* but not for *E. coli* when the LLA content increased.

The incorporation of 5% MCC in the 50% LLA-TPC film formed hydrogen bonds with the chitosan matrix, increasing the tensile strength of the TPC film. Strong interfacial adhesion between the chitosan film matrix and MCC particles was found. This addition also led to a decrease in the elongation at break of the TPC film. However, the tensile strength of the TPC/MCC films decreased again when the MCC content was higher than 5 wt%. All TPC/MCC composite films show antibacterial activities against both *S. aureus* and *E. coli*. The inhibition zone decreased significantly with 20 wt% MCC for both *S. aureus* and *E. coli*. The thermo-compressed TPC/MCC composite films, with their controllable properties, can be used in antibacterial food packaging applications. In addition, the research findings are expected to enable the development of eco-friendly and sustainable TPC/MCC composite products through other conventional melt processing techniques. Further research is necessary for the successful commercialization of TPC/MCC composite films as antibacterial food packaging. Barrier properties such as water vapor and oxygen permeability, as well as other antibacterial properties, need to be studied in conjunction with empirical food preservation testing and evaluation.

## Figures and Tables

**Table 1:** TGA results of TPC films.

LLA Content (wt%)	Char Residue at 800 °C (%)	*LLA-T_max_* (°C)	*CS-T_max_* (°C)
50	25.4	216	297
60	20.9	224	296
70	18.7	204, 238	305

**Table 2:** Tensile properties of TPC films.

LLA Content (wt%)	Maximum Tensile Strength (MPa)	Elongation at Break (%)	Young’s Modulus (MPa)
50	11.5 ± 1.4 ^c^	14.7 ± 2.1 ^a^	110.8 ± 8.2 ^c^
60	6.6 ± 0.5 ^b^	42.6 ± 2.8 ^b^	26.0 ± 3.4 ^b^
70	2.8 ± 0.4 ^a^	57.4 ± 4.6 ^c^	7.8 ± 1.2 ^a^

Values are presented as mean ± standard deviation (*n* = 5). Column values denoted by the letters (^a^, ^b^, and ^c^) exhibit significant differences (*p* < 0.05).

**Table 3:** Moisture content, surface wettability, and water dissolution of TPC films.

LLA Content (wt%)	Moisture Content (%)	Water Contact Angle (°)	Water Dissolution (%)
50	2.51 ± 0.14 ^a^	60.12 ± 2.54 ^b^	9.14 ± 0.43 ^b^
60	3.05 ± 0.09 ^b^	47.25 ± 3.64 ^a^	8.84 ± 0.77 ^b^
70	3.27 ± 0.20 ^b^	44.31 ± 2.86 ^a^	7.75 ± 0.61 ^a^

Values are presented as mean ± standard deviation (*n* = 3). Column values denoted by the letters (^a^ and ^b^) exhibit significant differences (*p* < 0.05).

**Table 4:** Film thickness, film opacity, and antibacterial activity of TPC films.

LLA Content (wt%)	Film Thickness (mm)	Film Opacity (mm^−1^)	Inhibition Zone (mm)	
			*S. aureus*	*E. coli*
50	0.69 ± 0.02 ^b^	1.32 ± 0.11 ^a^	4.0 ± 0.2 ^a^	1.9 ± 0.2 ^a^
60	0.55 ± 0.02 ^a^	1.39 ± 0.12 ^a, b^	5.3 ± 0.2 ^b^	2.0 ± 0.4 ^a^
70	0.53 ± 0.01 ^a^	1.48 ± 0.14 ^b^	6.0 ± 0.4 ^c^	1.8 ± 0.3 ^a^

Values are presented as mean ± standard deviation (*n* = 3). Column values denoted by the letters (^a^, ^b^, and ^c^) exhibit significant differences (*p* < 0.05).

**Table 5:** TGA results of TPC/MCC films. All films contain 50 wt% LLA, based on the weight of chitosan.

MCC Content (wt%)	Char Residue at 800 °C (%)	*LLA-T_max_* (°C)	*CS-T_max_* (°C)	*MCC-T_max_* (°C)
-	25.4	216	297	-
5	25.8	217	298	-
10	25.6	217	298	364
20	26.4	213	299	366

**Table 6:** Tensile properties of TPC/MCC films. All films contain 50 wt% LLA, based on the weight of chitosan.

MCC Content (wt%)	Maximum Tensile Strength (MPa)	Elongation at Break (%)	Young’s Modulus (MPa)
-	11.5 ± 1.4 ^a^	14.7 ± 2.1 ^c^	110.8 ± 8.2 ^a^
5	20.9 ± 1.2 ^b^	13.6 ± 2.2 ^b, c^	248.5 ± 10.5 ^b^
10	15.4 ± 1.8 ^c^	12.0 ± 1.8 ^b^	169.9 ± 8.4 ^c^
20	11.6 ± 1.5 ^a^	10.1 ± 1.2 ^a^	134.8 ± 8.8 ^d^

Values are presented as mean ± standard deviation (*n* = 5). Column values denoted by the letters (^a^, ^b^, ^c^, and ^d^) exhibit significant differences (*p* < 0.05).

**Table 7:** Moisture content, surface wettability, and water dissolution of TPC/MCC films. All films contain 50 wt% LLA, based on the weight of chitosan.

MCC Content (wt%)	Moisture Content (%)	Water Contact Angle (°)	Water Dissolution (%)
-	2.51 ± 0.14 ^a^	60.12 ± 2.54 ^d^	9.14 ± 0.43 ^c^
5	2.52 ± 0.19 ^a^	53.05 ± 1.85 ^c^	8.75 ± 0.38 ^b, c^
10	2.54 ± 0.03 ^a^	47.32 ± 3.82 ^b^	8.64 ± 0.56 ^b^
20	2.87 ± 0.10 ^b^	35.64 ± 3.56 ^a^	6.63 ± 0.15 ^a^

Values are presented as mean ± standard deviation (*n* = 3). Column values denoted by the letters (^a^, ^b^, ^c^, and ^d^) exhibit significant differences (*p* < 0.05).

**Table 8:** Film thickness, film opacity, and antibacterial activity of TPC/MCC films. All films contain 50 wt% LLA, based on the weight of chitosan.

MCC Content (wt%)	Film Thickness (mm)	Film Opacity (mm^−1^)	Inhibition Zone (mm)	
			*S. aureus*	*E. coli*
-	0.69 ± 0.02 ^a^	1.32 ± 0.11 ^a^	4.0 ± 0.2 ^b^	1.9 ± 0.2 ^b^
5	0.71 ± 0.02 ^a^	1.34 ± 0.14 ^a^	4.3 ± 0.2 ^b^	2.2 ± 0.3 ^b^
10	0.68 ± 0.03 ^a^	1.38 ± 0.18 ^a^	4.4 ± 0.3 ^b^	2.4 ± 0.5 ^b^
20	0.70 ± 0.05 ^a^	2.57 ± 0.15 ^b^	2.8 ± 0.4 ^a^	1.5 ± 0.3 ^a^

Values are presented as mean ± standard deviation (*n* = 3). Column values denoted by the letters (^a^ and ^b^) exhibit significant differences (*p* < 0.05).

## Data Availability

Data are contained within the article.

## Supplementary Materials

The following supporting information can be downloaded at: https://www.mdpi.com/article/10.3390/polym17182460/s1, Figure S1: (a) TG/DTG thermograms, (b) SEM image, and (c) XRD pattern of MCC powder.

## References

[1] Tripathi N., Misra M., Mohanty A.K. (2021). Durable polylactic acid (PLA)-based sustainable engineered blends and biocomposites: Recent developments, challenges, and opportunities. ACS Eng. Au.

[2] Andreeßen C., Steinbuchel A. (2019). Recent developments in non-biodegradable biopolymers: Precursors, production processes, and future perspectives. Appl. Microbiol. Biotechnol..

[3] Silva R.R.A., Marques C.S., Arruda T.R., Teixeira S.C., de Oliveira T.V. (2023). Biodegradation of polymers: Stages, measurement, standards and prospects. Macromol.

[4] Jariyasakoolroj P., Leelaphiwat P., Harnkarnsujarit N. (2020). Advances in research and development of bioplastic for food packaging. J. Sci. Food Agric..

[5] Kakadellis S., Harris Z.M. (2020). Don’t scrap the waste: The need for broader system boundaries in bioplastic food packaging life cycle assessment—A critical review. J. Clean. Prod..

[6] Zhao X., Cornish K., Vodovotz Y. (2020). Narrowing the gap for bioplastic use in food packaging: An update. Environ. Sci. Technol..

[7] Cheng J., Gao R., Zhu Y., Lin Q. (2024). Applications of biodegradable materials in food packaging: A review. Alex. Eng. J..

[8] Ambaye T.G., Vaccari M., Prasad S., Hullebusch E.D., Rtimi S. (2022). Preparation and applications of chitosan and cellulose composite materials. J. Environ. Manag..

[9] Strnad S., Zemljič L.F. (2023). Cellulose–chitosan functional biocomposites. Polymers.

[10] Jiang A., Patel R., Padhan B., Palimkar S., Galgali P., Adhikari A., Varga I., Patel M. (2023). Chitosan based biodegradable composite for antibacterial food packaging application. Polymers.

[11] Gao W., Mu B., Yang F., Li Y., Wang A. (2024). Green preparation of licorice flavonoids/ZnO/attapulgite nanocomposites for multifunctional chitosan-based food packaging films. LWT.

[12] Qu T., Wang X., Zhang F. (2025). Antibacterial food packaging with chitosan and cellulose blends for food preservation. Polymers.

[13] Dhalsamant K., Dalai A., Pattnaik F., Acharya B. (2025). Biodegradable carbohydrate-based films for packaging agricultural products—A review. Polymers.

[14] Ji Q., Su L., Boateng I.D., Li Z., Zhou C., Liu X., Ma Y. (2025). Preparation of chitosan/peanut shell nano-lignocellulose (CS/NLC) composite film and its preservation effect on cherry tomato and blueberry. Ind. Crops Prod..

[15] Matet M., Heuzey M.-C., Pollet E., Ajji A., Avérous L. (2013). Innovative thermoplastic chitosan obtained by thermo-mechanical mixing with polyol plasticizers. Carbohydr. Polym..

[16] Zhang Y., Liu B.-L., Wang L.-J., Deng Y.-H., Zhou S.-Y., Feng J.-W. (2019). Preparation, structure and properties of acid aqueous solution plasticized thermoplastic chitosan. Polymers.

[17] Dou X., Li Q., Wu Q., Duan L., Zhou S., Zhang Y. (2020). Effects of lactic acid and mixed acid aqueous solutions on the preparation, structure and properties of thermoplastic chitosan. Eur. Polym. J..

[18] Hanafy N.A., Leporatti S., El-Kemary M.A. (2019). Mucoadhesive hydrogel nanoparticles as smart biomedical drug delivery system. Appl. Sci..

[19] Cheng J., Gao M., Yang L., Zhang L., Zhu B. (2020). Coral-inspired “nanotentaclization” porous composite gel for efficient removal of Lead (II) from aqueous solution. Mater. Des..

[20] Yang J., Dahlstrom C., Edlund H., Lindman B., Norgren M. (2019). pH-responsive cellulose–chitosan nanocomposite films with slow release of chitosan. Cellulose.

[21] Di Liberto E.A., Dintcheva N.T. (2024). Biobased films based on chitosan and microcrystalline cellulose for sustainable packaging applications. Polymers.

[22] Rico M., Rodríguez-Llamazares S., Barral L., Bouza R., Montero B. (2016). Processing and characterization of polyols plasticized-starch reinforced with microcrystalline cellulose. Carbohydr. Polym..

[23] Deshmukh R.K., Tripathi S., Kumar P., Gaikwad K.K. (2025). Enhanced heat sealability and barrier performance of guar gum/polyvinyl alcohol based on biocomposite film reinforced with micro-fibrillated cellulose for packaging application. Polym. Bull..

[24] Schmid M., Reichert K., Hammann F., Stabler A. (2015). Storage time-dependent alteration of molecular interaction-property relationships of whey protein isolate-based films and coatings. J. Mater. Sci..

[25] Gao C., Pollet E., Avérous L. (2017). Innovative plasticized alginate obtained by thermo-mechanical mixing: Effect of different biobased polyols systems. Carbohydr. Polym..

[26] Hasheminya S.-M., Mokarram R.R., Ghanbarzadeh B., Hamishekar H., Kafil H.S., Dehghannya J. (2019). Development and characterization of biocomposite films made from kefiran, carboxymethyl cellulose and Satureja Khuzestanica essential oil. Food Chem..

[27] Tang Z., Fan F., Chu Z., Fan C., Qin Y. (2020). Barrier properties and characterizations of poly(lactic acid)/ZnO nanocomposites. Molecules.

[28] Bekbayeva L., Mun G.A., Yermukhambetova B.B., Negim E.-S., Irmukhametova G., Al Azzam K.M., Nechipurenko S.V., Efremov S.A., Yermaganbetov M., Samy M. (2025). Synthesis and characterization of biodegradable polymer blends based on chitosan. Polymers.

[29] Yasmeen S., Kabiraz M.K., Saha B., Qadir M.R., Gafur M.A., Masum S.M. (2016). Chromium (VI) ions removal from tannery effluent using chitosan-microcrystalline cellulose composite as adsorbent. Int. Res. J. Pure Appl. Chem..

[30] Zhai X., Zhang X., Ao H., Yin Y., Li X., Ren D. (2021). Preparation and characterization of whey protein isolate/chitosan/microcrystalline cellulose composite films. Packag. Technol. Sci..

[31] Benali Y., Mabrouki N., Agougui H., Jabli M., Majdoub H., Predoi D., Ciobanu S., Iconaru S.L., Ţălu S., Boughzala K. (2024). A new porous composite hydroxyapatite/chitosan/microcrystalline-cellulose: Synthesis, characterization and application to the adsorption of Eriochrome Black T. Polym. Bull..

[32] Chien N.V., Yen D.H., Phuong H.T., Trang P.Q., Diep N.T., Huy T.H., Phuong P.T., Tuyet P.A. (2024). Preparation of bioplastic materials based on thermoplastic chitosan and starch by melting mixing method. Vietnam J. Chem..

[33] Stroparo E.C., Mollinari K.C., de Souza K.V. (2018). Use of chitosan in the remediation of water from purification of biodiesel. Polímeros.

[34] Alrman K.H., Alhariri S., Al-Bakri I. (2024). Ultrafiltration membrane based on chitosan/adipic acid: Synthesis, characterization and performance on separation of methylene blue and reactive yellow-145 from aqueous phase. Heliyon.

[35] Xu J., Xia R., Zheng L., Yuan T., Sun R. (2019). Plasticized hemicelluloses/chitosan-based edible films reinforced by cellulose nanofiber with enhanced mechanical properties. Carbohydr. Polym..

[36] Zhang H., Yang S., Fang J., Deng Y., Wang D., Zhao Y. (2014). Optimization of the fermentation conditions of Rhizopus japonicus M193 for the production of chitin deacetylase and chitosan. Carbohydr. Polym..

[37] Weng R., Chen L., Lin S., Zhang H., Wu H., Liu K., Cao S., Huang L. (2017). Preparation and characterization of antibacterial cellulose/chitosan nanofiltration membranes. Polymers.

[38] Madian N.G., El-Ashmanty B.A., Abdel-Rahim H.K. (2023). Improvement of chitosan films properties by blending with cellulose, honey and curcumin. Polymers.

[39] Wadkin-Snaith D., Mulheran P.A., Johnston K. (2024). The impact of plasticisers on crystal nucleation, growth and melting in linear polymers. Polymer.

[40] Srinivasa P.C., Ramesh M.N., Kumar K.R., Tharanathan N. (2004). Properties of chitosan films prepared under different drying conditions. J. Food Eng..

[41] Mathew S., Brahmakumar M., Abraham T. (2006). Microstructural imaging and characterization of the mechanical, chemical, thermal, and swelling properties of starch-chitosan blend films. Biopolymers.

[42] Kaya M., Seyyar O., Baran T., Turkes T. (2014). Bat guano as new and attractive chitin and chitosan source. Front. Zool..

[43] Sun L., Sun J., Chen L., Niu P., Yang X., Guo Y. (2017). Preparation and characterization of chitosan film incorporated with thinned young apple polyphenols as an active packaging material. Carbohydr. Polym..

[44] Lin Y., Bilotti E., Bastiaansen C.W.M., Peijs T. (2020). Transparent semi-crystalline polymeric materials and their nanocomposites: A review. Polym. Eng. Sci..

[45] Qi L., Xu Z., Jiang X., Hu C., Zou X. (2004). Preparation and antibacterial activity of chitosan nanoparticles. Carbohydr. Res..

[46] Li K., Guan G., Zhu J., Wu H., Sun Q. (2019). Antibacterial activity and mechanism of a laccase-catalyzed chitosan–gallic acid derivative against Escherichia coli and Staphylococcus aureus. Food Control.

[47] Chandrasekaran M., Kim K.D., Chun S.C. (2020). Antibacterial activity of chitosan nanoparticles: A review. Processes.

[48] Guarnieri A., Triunfo M., Scieuzo C., Ianniciello D., Tafi E., Hahn T., Zibek S., Salvia R., De Bonis A., Falabella P. (2022). Antimicrobial properties of chitosan from different developmental stages of the bioconverter insect Hermetia illucens. Sci. Rep..

[49] Chang A.K.T., Frias R.R., Alvarez L.V., Bigol U.G., Guzman J.P.M.D. (2019). Comparative antibacterial activity of commercial chitosan and chitosan extracted from *Auricularia* sp.. Biocatal. Agric. Biotechnol..

[50] Wang W., Dong H., Chen Q., Chang X., Wang C., Miao C., Chen S., Chen L., Wang R., Ge S. (2024). Antibacterial efficacy of feline-derived lactic acid bacteria against Enteropathogenic *Escherichia coli*: A comprehensive in vitro analysis. Fermentation.

[51] Gao Z., Banan-Mwine Daliri E., Wang J., Liu D., Chen S., Ye X., Ding T. (2019). Inhibitory effect of lactic acid bacteria on foodborne pathogens: A review. J. Food Prot..

[52] Dai X., Xiong Z., Na H., Zhu J. (2014). How does epoxidized soybean oil improve the toughness of microcrystalline cellulose filled polylactide acid composites?. Compos. Sci. Technol..

[53] Lertprapaporn T., Manuspiya H., Laobuthee A. (2018). Dielectric improvement from novel polymeric hybrid films derived by polylactic acid/nanosilver coated microcrystalline cellulose. Mater. Today Proc..

[54] Othman N.A., Adam F., Yasin N.H.M. (2021). Reinforced bioplastic film at different microcrystalline cellulose concentration. Mater. Today Proc..

[55] Wang Z., Yu J., Zhang L., Zhou Y., Yang Y., Jin Y. (2017). Cellulose laurate ester aerogel as a novel absorbing material for removing pollutants from organic wastewater. Cellulose.

[56] Chang X.X., Mubarak N.M., Mazari S.A., Jatoi A.S., Ahmad A., Khalid M., Walvekar R., Abdullah E.C., Karri R.R., Siddiqui M.T.H. (2021). A review on the properties and applications of chitosan, cellulose and deep eutectic solvent in green chemistry. J. Ind. Eng. Chem..

[57] Yang J., Kwon G.-J., Hwang K., Kim D.-Y. (2018). Cellulose–chitosan antibacterial composite films prepared from LiBr solution. Polymers.

[58] Pongchaiphol S., Preechakun T., Raita M., Champreda V., Laosiripojana N. (2021). Characterization of cellulose–chitosan-based materials from different lignocellulosic residues prepared by the ethanosolv process and bleaching treatment with hydrogen peroxide. ACS Omega.

[59] Ting S.S., Hussain M., Yaacob N.D., Majib N.M., Hamimi S.N., See T.L., Kian T.W. (2024). Characterization and evaluation of polymer thin film composites from rice straw microcrystalline cellulose and mushroom chitosan. Malays. J. Microsc..

[60] Fouad H., Kian L.K., Jawaid M., Alotaibi M.D., Alothman O.Y., Hashem M. (2020). Characterization of microcrystalline cellulose isolated from Conocarpus fiber. Polymers.

[61] Asif M., Ahmed D., Ahmad N., Qamar M.T., Alruwaili N.K., Bukhari S.N.A. (2022). Extraction and Characterization of Microcrystalline Cellulose from Lagenaria siceraria Fruit Pedicles. Polymers.

[62] Hermawan D., Lai T.K., Jafarzadeh S.J., Gopakumar D.A., Hasan M., Owolabi F.A.T., Sri Aprilia N.A., Rizal S., Abdul Khalil H.P.S. (2019). Development of seaweed-based bamboo microcrystalline cellulose films intended for sustainable food packaging applications. BioResources.

[63] Désiré A.Y., Charlemagn N., Claver K.D., Achille T.F., Marianne S. (2021). Starch-based edible films of improved cassava varieties Yavo and TMS reinforced with microcrystalline cellulose. Heliyon.

[64] Lubis R., Wirjosentono B., Eddyanto Septevani A.A. (2020). Preparation, characterization and antimicrobial activity of grafted cellulose fiber from durian rind waste. Colloids Surf. A Physicochem. Eng. Asp..

[65] Sun Y., Wang J., Li D., Cheng F. (2024). The recent progress of the cellulose-based antibacterial hydrogel. Gels.

[66] Hu P., Lai A., Zhou S. (2025). Preparation and properties of antibacterial PVA@MCC composite membrane assisted by ionic liquids and DMSO. Chin. J. Chem. Eng..

