# Supplementary figures and images for: Thermo-Compression of Thermoplastic Chitosan Films Reinforced with Microcrystalline Cellulose for Antibacterial Food Packaging Application
Source: Polymers (Basel). 2025 Sep 11;17(18):2460. doi: 10.3390/polym17182460 (PMC12473501; doi:10.3390/polym17182460)

1

## Supplementary Material

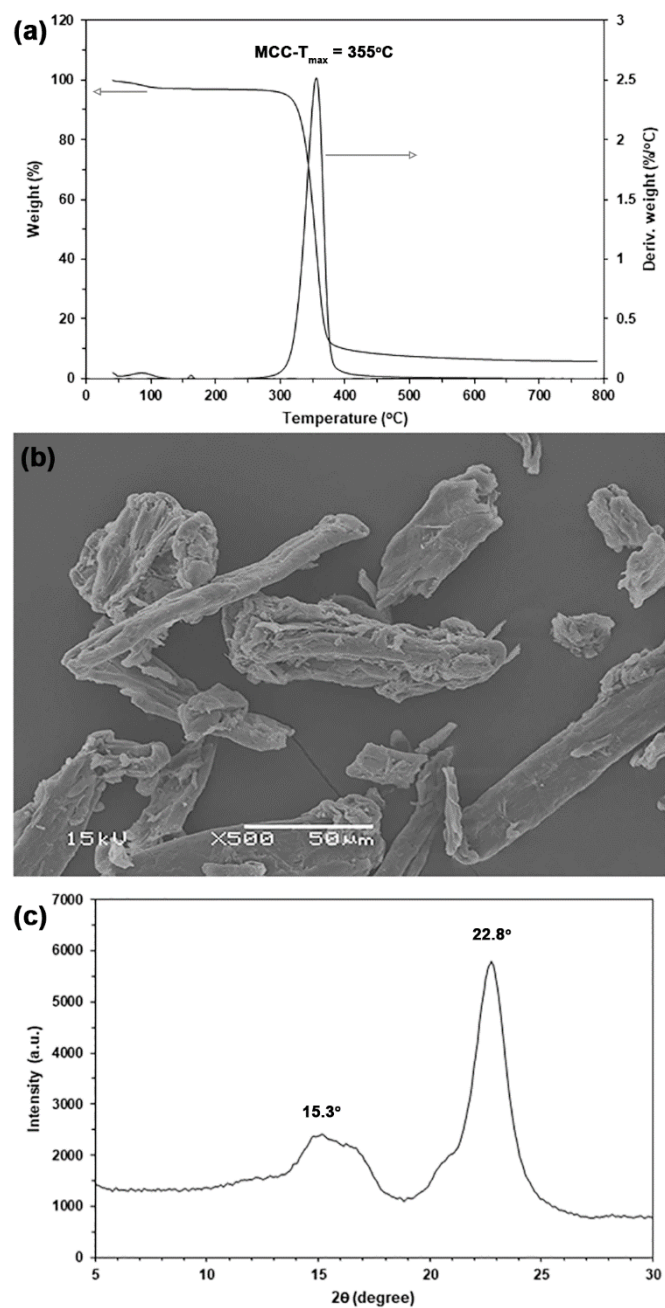

2

3

Fig. S1. (a) TG/DTG thermograms, (b) SEM image, and (c) XRD pattern of MCC powder.

Supplement: Supplementary file 1 [file polymers-17-02460-s001.zip › polymers-3828673-supplementary.pdf]
